# Do subclinical anxious and depressive symptoms affect facial emotion recognition? An exploratory investigation

**DOI:** 10.1590/1980-5764-DN-2025-0304

**Published:** 2025-10-27

**Authors:** Agnes Stéphanie da Silva, Aline Silva de Miranda, Maíra Glória de Freitas Cardoso, Luciano Inácio Mariano, Vinicius Sousa Pietra Pedroso, Antônio Lúcio Teixeira, Leonardo Cruz de Souza

**Affiliations:** 1 Universidade Federal de Minas Gerais, Programa de Pós-Graduação em Neurociências, Belo Horizonte MG, Brazil.; 2 Universidade Federal de Minas Gerais, Departamento de Morfologia, Belo Horizonte MG, Brazil.; 3 Pontifícia Universidade Católica de Minas Gerais, Departamento de Psicologia, Belo Horizonte MG, Brazil.; 4 Pontifícia Universidade Católica de Minas Gerais, Departamento de Medicina, Belo Horizonte MG, Brazil.; 5 University of Texas Health Science Center at Houston, Neuropsychiatry Program, Department of Psychiatry and Behavioral Sciences, McGovern Medical School, Houston TX, United States of America.; 6 Universidade Federal de Minas Gerais, Departamento de Clínica Médica, Belo Horizonte MG, Brazil.

**Keywords:** Facial Recognition, Anxiety, Depression, Neuropsychological Tests, Social Cognition, Reconhecimento Facial, Ansiedade, Depressão, Testes Neuropsicológicos, Cognição Social

## Abstract

Emotion recognition is a key component of social cognition. Symptoms of depression and anxiety may modulate this ability.

**Objective:**

To investigate the effect of subclinical depressive and anxiety symptoms on performance in a facial emotion recognition test.

**Methods:**

A total of 203 participants (109 females and 94 males; mean age 48.8±19 years; mean schooling 9.3±5.1 years) without current or past history of psychiatric or neurological disorders were enrolled. None had abnormal scores on the anxiety or depression subscales of the Hospital Anxiety and Depression (HAD) Scale. All participants (N=203) underwent the Mini-Mental State Exam (MMSE) and the Facial Emotion Recognition Test (FERT), which assesses recognition of: anger, disgust, fear, happiness, sadness, surprise, and neutral expressions. Multivariate analyses included HAD-Anxiety, HAD-Depression, MMSE, age, and schooling as covariates.

**Results:**

Age, schooling, and MMSE were significantly correlated with FERT scores. A significant negative correlation was found between FERT-Total and HAD-Depression (r=-0.22; p<0.002), and between FERT-Fear and HAD-Depression (r=-0.31; p<0.0001). No other significant correlations were observed between FERT scores and psychiatric measures. Anxiety and depression scores were not retained in the final multivariate models, except for a negative correlation between HAD-Anxiety and FERT-Fear.

**Conclusion:**

Subclinical anxious and depressive symptoms have a modest effect on FERT.

## RESUMO.

O reconhecimento de emoções é um componente fundamental da cognição social. Sintomas de depressão e ansiedade podem modular essa habilidade.

**Objetivo:**

Investigar o efeito de sintomas depressivos e ansiosos subclínicos no desempenho em um teste de reconhecimento de emoções faciais.

**Métodos:**

Um total de 203 participantes (109 do sexo feminino e 94 do sexo masculino; idade média de 48,8±19 anos; escolaridade média de 9,3±5,1 anos) sem histórico atual ou prévio de transtornos psiquiátricos ou neurológicos foram incluídos. Nenhum apresentou escores anormais nas subescalas de ansiedade ou depressão da Escala Hospitalar de Ansiedade e Depressão (Hospital Anxiety and Depression Scale – HAD). Todos os participantes (N=203) realizaram o Mini Exame do Estado Mental (Mini-Mental State Examination – MMSE) e o Teste de Reconhecimento de Emoções Faciais (Facial Emotion Recognition Test – FERT), que avalia o reconhecimento das expressões de: raiva, nojo, medo, alegria, tristeza, surpresa e neutra. As análises multivariadas incluíram como covariáveis: HAD-Ansiedade, HAD-Depressão, MMSE, idade e escolaridade.

**Resultados:**

Idade, escolaridade e MMSE apresentaram correlação significativa com os escores do FERT. Foi observada uma correlação negativa significativa entre o FERT-Total e a HAD-Depressão (r=-0,22; p<0,002), bem como entre o FERT-Medo e a HAD-Depressão (r=-0,31; p<0,0001). Nenhuma outra correlação significativa foi observada entre os escores do FERT e as medidas psiquiátricas. As pontuações de ansiedade e depressão não foram confirmadas nos modelos multivariados finais, com exceção de uma correlação negativa entre HAD-Ansiedade e FERT-Medo.

**Conclusão:**

Sintomas ansiosos e depressivos subclínicos têm um efeito modesto no FERT.

## INTRODUCTION

 Emotion recognition is a core ability for adaptive and social behaviors. Seminal studies by Charles Darwin (1809–1882) demonstrated the ubiquitous presence of this ability across different species. Paul Ekman (b. 1934) founded the modern study of facial emotion recognition by demonstrating its universality across cultures^1^. Ekman postulated a set of so-called primary emotions (happiness, sadness, surprise, disgust, anger, and fear) and provided a standardized framework for assessing these emotions in research and clinical settings^2^. 

 More recently, experimental and clinical studies have explored factors that influence facial emotion identification. Age, gender, education, and cultural background may interfere with performance in emotion recognition tasks^3-6^. From a clinical perspective, it has also been demonstrated that various neurodegenerative diseases impair emotion recognition, such as behavioral variant frontotemporal dementia^7,8^. 

 Primary psychiatric disorders may also affect the processing of facial emotions. Deficits in emotion recognition have been described in major depressive disorder (MDD) and anxiety disorders. Notwithstanding, the role of subclinical psychiatric symptoms in emotion recognition remains unclear. Subclinical symptoms of depression or anxiety refer to a condition in which an individual presents psychiatric symptoms but does not meet the full diagnostic criteria for a depressive or anxiety disorder, respectively^9,10^. These conditions may be referred to as "subsyndromal depression"^11,12^ or "subsyndromal anxiety"^13,14^ and are clinically relevant, as individuals with these symptoms are at higher risk of developing a full syndrome^12,13,15^ and tend to exhibit greater functional impairment^11^. Subclinical psychiatric symptoms may introduce biases in the identification of facial expressions^16^ and, in turn, may contribute to deficits in emotion recognition. For instance, anxiety may influence the processing of emotional facial expressions, with biases related to both attention and interpretation^17,18^. Moreover, biases in the processing of emotional facial expressions have also been identified in MDD, even after remission of a depressive episode^19^. 

 Studies on this topic are divided between those that assess emotion recognition according to emotional intensity and those that consider only emotional valence. Symptoms of depression and state anxiety have been associated with difficulties in evaluating neutral faces among non-clinical individuals, while trait anxiety has been linked to improved recognition of happiness, anger, and fear. Trait anxiety was associated with better recognition of anger, fear, and happiness. On the other hand, participants with high depression scores were more likely to attribute fear to neutral faces^20^. Moreover, depressivity modulates the recognition of happiness^21,22^. For individuals with anxiety, a higher emotional intensity is required to identify happy and sad facial expressions compared to those with non-anxious depression^20^. 

 This study aimed to investigate the effect of subclinical symptoms of anxiety and depression on performance in a facial emotion recognition test. It was hypothesized that subclinical anxiety and depressive symptoms would affect performance on the test through a negative bias. 

## METHODS

 This study was approved by the Local Ethics Committee of Universidade Federal de Minas Gerais (CAA17850513.2.00005149). All participants provided written informed consent prior to participation. 

 A total of 203 individuals participated in the study (109 females and 94 males; mean age 48.8±19 years [15–86 years]; mean education 9.3±5.1 years [0–22 years], recruited for a larger transcultural study^3^. Demographic data for the sample are presented in Table 1. 

**Table 1:** Demographic data of the sample (median±SD).

Characteristics	
Total number of participants	203
Sex (Male/Female)	94/109
Age (years)	48.8±19.2
Schooling (years of education)	9.3±5.1

Abbreviation: SD, standard deviation.

 All participants underwent a standardized interview focusing on neurological and psychiatric history. Participants were recruited from the community on a voluntary basis under the following inclusion criteria:

- Absence of cognitive complaints;
- normal performance on the Mini-Mental State Examination (MMSE)^23^, based on education-adjusted norms for the Brazilian population.
- The following exclusion criteria were adopted:
- past or current diagnosis of severe psychiatric disorders (*e.g.*, schizophrenia, bipolar disorder);
- past or current diagnosis of neurological diseases (*e.g.*, stroke, epilepsy, multiple sclerosis, dementia);
- medical history of neurosurgical procedures;
- use of medications that may interfere with cognitive performance (*e.g.*, benzodiazepines; antipsychotics);
- scores higher than 9 (out of 21) on either the anxiety or depression subscales of the Hospital Anxiety and Depression (HAD) Scale, indicating clinically relevant anxiety or depression^24^.

All participants (N=203) completed the Brazilian version of the Facial Emotion Recognition Test (FERT), as previously described^3^. This version has been validated for use in Brazil and has been successfully employed in clinical research^6,25-27^. FERT comprises 35 pictures from Ekman’s portfolio and assesses the recognition of the following emotions: anger, disgust, fear, happiness, sadness, surprise, and neutral. 

### Statistical analysis

 All statistical analyses were performed using the Statistical Package for Social Sciences (SPPSS, version 22). Descriptive statistics were used to characterize the sample. The assumption of normality was verified using the Kolmogorov–Smirnov test. As the variables did not follow a Gaussian distribution, non-parametric tests were employed. Spearman’s correlation test was used to examine associations between FERT scores (total and subscores for each emotion) and HAD-Anxiety and HAD-Depression scores. Bonferroni correction was applied for multiple comparisons, and the level of significance (α) was set at 0.003. 

 A linear regression model (Enter Method) was used to investigate the relationship between FERT scores (FERT-Total, FERT-Anger, FERT-Disgust, FERT-Fear, FERT-Happiness, FERT-Sadness, FERT-Surprise, and FERT-Neutral) and the following potential predictors: schooling (years of education), MMSE, HAD-Anxiety and HAD-Depression. Logarithmic transformation of FERT scores were used as dependent variables, while age, schooling, MMSE, HAD-anxiety, and HAD-depression were included as independent variables. 

## RESULTS

 Participants had a mean score of 3.12 (SD=2.02) on the HAD-Anxiety subscale and 2.46 (SD=2.04) on the HAD-Depression subscale. The mean total score on FERT was 26.34 (SD=4.44), and the average scores for each emotion were: 4.87 (SD=0.36) for happiness, 3.96 (SD=1.11) for surprise, 3.77 (SD=1.08) for disgust, 2.49 (SD=1.32) for fear, 3.40 (SD=1.05) for anger, 3.64 (SD=1.09) for sadness, and 4.2 (SD=1.04) for neutral. 

 Correlations between FERT scores and HAD-Anxiety and HAD-Depression scores were examined using Spearman’s test (Table 2). A significant negative correlation was found between FERT-Total and HAD-Depression (r=-0.22; p<0.002), as well as between FERT-Fear and HAD-Depression (r=-0.31; p<0.0001). No other significant correlations were observed between FERT scores and psychiatric scores. 

**Table 2:** Results for correlations analyses (Spearman’s correlation test) between psychiatric scores (anxiety and depression from the Hospital Anxiety and Depression [HAD] Scale) and the Facial Emotion Recognition Test.

		Happiness	Surprise	Disgust	Fear	Anger	Sadness	Neutral	Total
Anxiety	r	0.141	0.136	0.028	-0.200	-0.065	0.068	0.045	-0.057
	p	<0.045	<0.053	<0.687	<0.004	<0.355	<0.338	<0.528	<0.418
Depression	r	0.005	0.012	-0.087	-0.308	-0.185	-0.074	-0.004	-0.221
	p	<0.946	<0.866	<0.218	<0.0001*	<0.008	<0.293	<0.952	<0.002*

Notes: *Significant after Bonferroni correction (p<0.003); r: Spearman’s coefficient; p: significance level.

 Results of the multivariate analyses are presented in Table 3. Linear regression showed that FERT-Total was positively correlated with schooling and MMSE, and negatively correlated with age. The final multiple regression model, which included age, schooling, and MMSE, was statistically significant (R^2^=0.59, *F*(3, 185)=89.79, p<0.0001). HAD-Depression and HAD-Anxiety did not contribute to the final model. 

**Table 3:** Summary statistics and results from the regression analysis.

Model	Variable	B	Standard error	Beta	p-value
FERT-Anger	Education	0.278	0.036	0.495	<0.0001
FERT-Disgust	MMSE	1.551	0.181	0.521	<0.0001
FERT-Fear	MMSE	1.312	0.339	0.287	<0.0001
	Age	-0.257	0.078	-0.240	<0.001
	HAD-Anxiety	-0.153	0.057	-0.187	<0.008
FERT-Happiness	MMSE	0.431	0.045	0.557	<0.0001
FERT-Neutral	MMSE	0.973	0.329	0.243	<0.004
	Education	0.225	0.43	0.434	<0.0001
FERT-Sadness	MMSE	1.986	0.189	0.597	<0.0001
FERT-Surprise	MMSE	1.741	0.399	0.392	<0.0001
	Education	0.114	0.051	0.201	<0.027
FERT-Total	Age	-0.044	0.018	-0.120	<0.017
	Education	0.116	0.019	0.425	<0.0001
	MMSE	0.745	0.148	0.350	<0.0001

Abbreviations: FERT, Facial Emotion Recognition Test; MMSE, Mini-Mental State Exam; HAD, Hospital Anxiety and Depression Scale.

 Each individual FERT emotion score was then analyzed separately. FERT-Happiness was positively correlated with MMSE. The final model for FERT-Happiness include only MMSE (R^2^=0.31, *F*(1, 200)=89.79, p<0.0001). FERT-Surprise was positively correlated with both MMSE and schooling; these variable were retained in the final statistically significant model (R^2^=0.31, *F*(2, 186)=41.74, p<0.0001). Again, HAD-Depression and HAD-Anxiety did not contribute to the final regression models. The final model for FERT-Disgust was also statistically significant and included only MMSE as a predictor (R^2^=0.27, *F*(1, 198)=73.72, p<0.001). Similarly, the final significant model for FERT-Sadness retained only MMSE (R^2^=0.36, *F*(1, 200)=110.85, p<0.0001). Linear regression showed that FERT-Fear was significantly positively correlated with MMSE, and negatively correlated with age and HAD-Anxiety. The final multiple regression model including these variables was statistically significant (R^2^=0.20, *F*(3, 171)=14.57, p<0.0001). The final model fort FERT-Anger was also statistically significant and included only schooling as a predictor (R^2^=0.25, *F*(1, 185)=59.98, p<0.0001). Finally, FERT-Neutral was positively correlated with both MMSE and schooling, and the final model including these variables was statistically significant (R^2^=0.40, *F*(2, 185)=61.62, p<0.0001). 

## DISCUSSION

 This exploratory study investigated whether subclinical symptoms of anxiety and depression modulate facial emotion recognition in a population of aged subjects. Contrary to our hypothesis, there was only a modest effect of subclinical psychiatric symptoms on performance in FERT. More specifically, mild negative correlations were found between HAD-Depression and both FERT-Total and FERT-Fear scores. 

 Previous studies have demonstrated a negative bias in the processing of facial emotions in MDD^21,22,28-30^. Patients with MDD tend to interpret emotions more negatively; ambiguous or neutral facial expressions are more likely to be perceived as negative and are more frequently recognized as sad faces^28^. Additionally, individuals with MDD often require greater emotional intensity to accurately identify happy expressions^31^. This processing dysfunction may impair interpersonal interaction and functioning^19^. Interestingly, such biases in the processing of emotional facial expressions are observed even after recovery from a depressive episode and are considered a risk factor for recurrence^19^. Regarding anxiety disorders, some studies suggest that anxiety is associated with an increased ability to identify fearful expressions^18^, while others do not support this association^20^. 

 Overall, anxiety and depression scores were not retained in the final multivariate models, except for a negative correlation between HAD-Anxiety and FERT-Fear. Accordingly, contrariwise to the original hypothesis, subclinical anxiety and depressive symptoms do not appear to significantly influence emotion recognition. 

 These findings are consistent with previous evidence that age and education modulate performance in emotion recognition task^3-5^. It is well recognized that emotion recognition abilities decline with age^5^. This decline may be attributed to general cognitive impairment resulting from age-related pathological changes in brain regions involved in emotional processing^5^. In line with this view, significant correlations were observed between MMSE and FERT scores, indicating that general cognitive efficiency influences emotion recognition abilities. 

 The limitations of this study must be acknowledged. The sample was derived from a normative database^3^, and none of the participants exhibited marked anxious or depressive symptoms. Moreover, the original study^3^ was not specifically designed to select individuals with subclinical symptoms of anxiety or depression. It is therefore possible that the sample included subjects with minimal symptomatology ("ground effect"), limiting the potential for meaningful correlations between psychiatric symptoms and FERT performance. This may explain the modest effects observed. Additionally, characteristics of FERT itself may have contributed to these mild correlations. Indeed, FERT may lack sensitivity to detect subtle deficits in individuals with subclinical psychiatric symptoms. More modern tools, incorporating dynamic facial expression recognition and varying levels of emotional intensity, may be more suitable for assessing patients with milder psychiatric symptoms. Therefore, more studies are warranted to address the effect of subclinical symptoms on emotional processing. Besides these limitations, this exploratory study shed light on factors that may influence performance in facial emotion recognition tasks. 

## Data Availability

The datasets generated and/or analyzed during the current study are not publicly available due to [ethical/legal/privacy] restrictions but are available from the corresponding author upon reasonable request.

## References

[1] Ekman P, Friesen WV, O’Sullivan M, Chan A, Diacoyanni-Tarlatzis I, Heider K (1987). Universals and cultural differences in the judgments of facial expressions of emotion. J Pers Soc Psychol.

[2] Ekman P (1993). Facial expression and emotion. Am Psychol.

[3] de Souza LC, Bertoux M, Vaz de Faria AR, Corgosinho LTS, Prado ACA, Barbosa IG (2018). The effects of gender, age, schooling, and cultural background on the identification of facial emotions: a transcultural study. Int Psychogeriatr.

[4] Engelmann JB, Pogosyan M (2013). Emotion perception across cultures: the role of cognitive mechanisms. Front Psychol.

[5] Ruffman T, Henry JD, Livingstone V, Philips LH (2008). A meta-analytic review of emotion recognition and aging: implications for neuropsychological models of aging. Neurosci Biobehav Rev.

[6] Quesque F, Coutrot A, Cox A, Souza LC, Baez S, Cardona JF (2022). Does culture shape our understanding of others’ thoughts and emotions? An investigation across 12 countries. Neuropsychology.

[7] Bertoux M, de Souza LC, Sarazin M, Funkiewiez A, Dubois B, Hornberger M (2015). How preserved is emotion recognition in Alzheimer disease compared with behavioral variant frontotemporal dementia?. Alzheimer Dis Assoc Disord.

[8] de Souza LC, Bertoux M, Radakovic R, Hornberger M, Mariano LI, Resende EPF (2022). I’m looking through you: Mentalizing in frontotemporal dementia and progressive supranuclear palsy. Cortex.

[9] Lyu C, Lyu X, Gong Q, Gao B, Wang Y (2024). Neural activation signatures in individuals with subclinical depression: A task-fMRI meta-analysis. J Affect Disord.

[10] Brebion G, Nunez C, Lombardini F, Senior C, Laforga AMS, Siddi S (2021). Subclinical depression and anxiety impact verbal memory functioning differently in men and women -an fMRI study. J Psychiatr Res.

[11] Lyness JM, Kim J, Tu X, Conwell Y, King DA, Caine ED (2007). The clinical significance of subsyndromal depression in older primary care patients. Am J Geriatr Psychiatry.

[12] Lyness JM (2008). Naturalistic outcomes of minor and subsyndromal depression in older primary care patients. Int J Geriatr Psychiatry.

[13] Volz HP, Saliger J, Kasper S, Möller H-J, Seifritz E (2022). Subsyndromal generalised anxiety disorder: operationalisation and epidemiology - a systematic literature survey. Int J Psychiatry Clin Pract.

[14] Miloyan B, Byrne GJ, Pachana NA (2015). Threshold and subthreshold generalized anxiety disorder in later life. Am J Geriatr Psychiatry.

[15] Corpas J, Gilbody S, McMillan D (2022). ognitive, behavioural or cognitive-behavioural self-help interventions for subclinical depression in older adults: A systematic review and meta-analysis. J Affect Disord.

[16] Kennedy M, Simcock G, Jamieson D, Hermens DF, Lagopoulos J, Shan Z (2021). Elucidating the neural correlates of emotion recognition in children with sub-clinical anxiety. J Psychiatr Res.

[17] Bar-Haim Y, Lamy D, Pergamin L, Bakermans-Kranenburg MJ, van IJzendoorn MH (2007). Threat-related attentional bias in anxious and nonanxious individuals: a meta-analytic study. Psychol Bull.

[18] Surcinelli P, Codispoti M, Montebarocci O, Rossi N, Baldaro B (2006). Facial emotion recognition in trait anxiety. J Anxiety Disord.

[19] LeMoult J, Joormann J, Sherdell L, Wright Y, Gotlib IH (2009). Identification of emotional facial expressions following recovery from depression. J Abnorm Psychol.

[20] Cooper RM, Rowe AC, Penton-Voak IS (2008). The role of trait anxiety in the recognition of emotional facial expressions. J Anxiety Disord.

[21] Krause FC, Linardatos E, Fresco DM, Moore MT (2021). Facial emotion recognition in major depressive disorder: A meta-analytic review. J Affect Disord.

[22] Monferrer M, Garcia AS, Ricarte JJ, Montes MJ, Fernández-Caballero A, Fernández-Sotos P (2023). Facial emotion recognition in patients with depression compared to healthy controls when using human avatars. Sci Rep.

[23] Brucki SM, Nitrini R, Caramelli P, Bertolucci PHF, Okamoto IH (2003). [Suggestions for utilization of the mini-mental state examination in Brazil]. Arq Neuropsiquiatr.

[24] Botega NJ, Bio MR, Zomignani MA, Garcia C, Pereira WAB (1995). [Mood disorders among inpatients in ambulatory and validation of the anxiety and depression scale HAD]. Rev Saude Publica.

[25] Moura MVB, Mariano LI, Teixeira AL, Caramelli P, Souza LC (2021). Social cognition tests can discriminate behavioral variant frontotemporal dementia From Alzheimer’s disease independently of executive functioning. Arch Clin Neuropsychol.

[26] Martins MI, Cardoso FEC, Caramelli P, Mariano LI, Rocha NP, Jaeger A (2024). Hearts and minds: emotion recognition and mentalizing in Parkinson’s disease and progressive supranuclear palsy. Arch Clin Neuropsychol.

[27] Barbosa IG, da Leite FMC, Bertoux M, Guimarães HC, Mariano LI, Gambogi LB (2023). Social cognition across bipolar disorder and behavioral variant frontotemporal dementia: an exploratory study. Braz J Psychiatry.

[28] Bourke C, Douglas K, Porter R (2010). Processing of facial emotion expression in major depression: a review. Aust N Z J Psychiatry.

[29] Liu WH, Huang J, Wang LZ, Gong Q-Y, Chan RCK (2012). Facial perception bias in patients with major depression. Psychiatry Res.

[30] Münkler P, Rothkirch M, Dalati Y, Schmack K, Sterzer P (2015). Biased recognition of facial affect in patients with major depressive disorder reflects clinical state. PLoS One.

[31] Berg HE, Ballard ED, Luckenbaugh DA, Nugent AC, Ionescu DF, Zarate CA (2016). Recognition of emotional facial expressions in anxious and nonanxious depression. Compr Psychiatry.

